# Scheduled, cancelled, rescheduled: navigating educational supervision in residency training

**DOI:** 10.5116/ijme.687b.7d22

**Published:** 2025-07-28

**Authors:** Cecilie Normann Birkeli, Karin Isaksson Rø, Monika Kvernenes

**Affiliations:** 1 Institute for Studies of the Medical Profession, Oslo, Norway; 2 Center for Medical Education and Department of Clinical Medicine, Faculty of Medicine, University of Bergen, Norway

**Keywords:** Educational supervision, educational supervisor, medical resident, residency training, postgraduate medical educa-tion, competency-based medical education, hospital training, medical specialist training

## Abstract

**Objectives:**

This study aims to enhance our understanding of how educational supervision operates from the perspective of medical residents, and how they engage with it within the context of implementing competency-based medical education.

**Methods:**

We conducted a qualitative research study following the principles of grounded theory methodology. Participants were recruited from national residency training courses. Data was collected using an electronically distributed questionnaire with open-ended questions, which invited respondents to share their experiences with educational supervision. 96 written narrative responses were applicable for analysis.

**Results:**

We identified three categories indicative of residents’ experiences with educational supervision: I) Access to educational supervision, II) Links between quality of educational supervision and organisational facilitation, and III) Pushbacks to educational supervision and how residents cope with pushbacks. Residents’ experiences varied significantly. When educational supervision was well-organised and available, residents managed to express how educational supervision enhanced their education. However, many residents struggled to access educational supervision (ES).

**Conclusion:**

When educational supervision is integrated into clinical practice, residents perceive its benefit to their education. Conversely, inadequate organisation of educational supervision forces residents to expend significant effort to ensure meetings occur. Amidst the implementation of competency-based medical education, residents risk being left with the individual responsibility to initiate and sustain educational supervision, which in turn places an undue burden on trainees to navigate repeated pushbacks, and workplace cultures that devalues educational support. Further research is needed to explore the affordances relevant for different medical specialties, and observational studies are much needed as a complement to self-reported data.

## Introduction

Educational supervision (ES) is an important support structure for hospital medical residents in specialist training. ES has been described as: ‘regular supervision taking place in the context of a recognized training, in order to establish learning needs and review progress’.^1^ An educational supervisor is responsible for coordinating and overseeing learning progression and engaging in supportive and reflective conversation with the trainee.^2^

Educational supervisors are responsible for facilitating learning and for assessing the performance of the supervisee.^1^ Parallel to the support from educational supervisors, residents are typically supervised by different clinicians who work with them on a day-to-day basis. This is referred to as clinical supervision and includes practical supervision during ward rounds, treatment related advice, and reflective case-based discussions. ES is, however, not limited to clinical work but includes all aspects of the trainee’s role.^1^^,^^2^

The distinction between the two support functions, ES and CS are often misunderstood, partly due to unclear definitions and partly because the same person may occupy both roles in different contexts, depending on the task at hand.^3^ The need to define these roles and assign specific responsibilities to them arose more clearly with the implementation of competency-based medical education (CBME).

Over the past 15 years, CBME has been implemented in several countries to the extent that it is now referred to as a global movement.^3^ CBME has been defined as: ‘an outcomes-based approach to the design, implementation, assessment, and evaluation of medical education programs, using an organising framework of competencies’ (p. 641).^4^ It adopts a learner-centred approach, focuses on specific competencies and employs competence-dependent rather than time-dependent certifications.^4^ These competencies need to be monitored during residents’ course of training to ensure individual progression.

Transitioning from a traditional regime to CBME directly impacts the functioning and perceptions of postgraduate medical education delivery.^5^ ES has become more prominent due to the need for monitoring and supporting the trainee’s progression more closely.^6^ Medical specialist training is conducted mainly at work and benefits from the unique learning opportunities real life practice offers. However, educational processes can be challenged by traditions, structures, and organisational factors embedded in the workplace.^7^ ES requires that time be set aside away from the clinic, and this can be difficult to achieve in a hectic working environment where patient care is the main priority. ES offers important support for residents in following the workplace curriculum. Furthermore, lack of well-functioning ES may lead to reduced learning outcomes, unproductive learning strategies, stress, and reduced well-being amongst residents.^8^

A study in the UK found that many trainees felt they were offered limited ES, and study participants reported that approximately half of the ES meetings lasted only 10–20 minutes.^9^ Another study on specialist registrars’ views of ES and how it could be improved showed that more than a third of the trainees rated the ES they had received as being closer to ‘a complete waste of time, than to excellent’.^10^ One of the main challenges was the perceived lack of commitment by individual educational supervisors.

The importance of a safe learning environment that includes a constructive relationship between supervisor and resident has been pointed out in earlier studies.^11^^-^^13^While enhancing ES is a key component of CBME implementation, residents’ have described tensions between feedback focused on growth and development, versus assessment of the residents’ progress.^14^ This tension can have a negative impact on the ES setting. Moreover, studies indicate that residents hold diverse perceptions and varying levels of understanding regarding the foundational framework of implementing CBME.^15^ Another study found that, although residents anticipate improved assessment and feedback, as well as earlier identification of difficulties in training with ES, they also fear disadvantages such as logistical challenges, the inability of attending physicians to provide support, and a resultant lack of appropriate feedback and assessment.^16^

Adding to these challenges, previous research has shown that the discourse suffers from a lack of common understanding about what ES entails in clinical practice, and that the concept of ES is poorly understood.^3^^,^^17^ There is no universal definition of ES, terms such as ‘educational supervision’, ‘mentoring’ and ‘feedback’ are used interchangeably, and the content of ES practices varies within different contexts.^3^ ES has been introduced to help residents in their specialist training, however there is little research on how residents themselves experience ES. Questions such as ‘How is ES conducted in the workplace setting?’ and ‘Is ES considered useful from the residents perspective?’ are still pending. If the quality of residency training is to be improved, we need to progress beyond establishing guidelines and apply knowledge about how residents engage with ES in practice. Therefore, this study aims to investigate how residents perceive educational supervision in their training at the hospital by conducting a qualitative survey with open-ended questions. The research question we seek to answer is: How do hospital residents engage with educational supervision in the context of CBME implementation?

## Methods

### Study design and participants

This study employs a qualitative grounded theory research design to explore residents’ experiences with ES. Given the limited previous research on ES practices,^3^ grounded theory offers a systematic yet flexible approach to generate theory directly from participants’ perspectives. Data were gathered through open-ended narrative responses via an electronic questionnaire, which enabled both in-depth description and breadth by capturing diverse experiences across heterogeneous workplaces. This design supports inductive analysis without imposing preconceived categories, allowing theory to emerge organically from the data.^18^^,^^19^

Participants were recruited among residents in internal medicine, surgery, and anaesthesiology, which are the main specialties within somatic hospital residency training. In Norway, approximately 1050, 300, and 300 residents are enrolled in these residency programs, respectively. We began with convenience sampling to reach a diverse and relevant group of residents. This was done by recruiting participants from national theoretical courses for residents within these specialties. In the courses, the first author gave a short presentation of the purpose of the study and handed out invitations to participate which included an open link or QR code to the questionnaire. The responses were anonymous. Participants were encouraged to share the link with other residents so that we could collect as many responses as possible. This approach enabled us to gather rich initial data from varied contexts, which is consistent with grounded theory’s iterative and flexible nature. As our initial analysis emerged, we sought additional data amongst surgical residents to more fully understand the significance of workplace characteristics in surgery. This approach reflects the principles of theoretical sampling, where data collection is guided by the evolving analysis.

This study has been submitted for assessment and was exempted from approval by REK (Regional committee for medical health research ethics) since no health-related data were gathered.

### Study setting

The study was conducted in Norway, where the postgraduate medical education system has recently been reformed towards adopting CMBE principles: A new specialisation system was introduced for medical interns in 2017, and for medical residents in 2019, placing a greater emphasis on learning outcomes, continuing assessment and ES.^20^ Residency training for most specialties takes place in hospitals that have been formally approved as educational institutions. The national specialist regulation mandates that the total duration of the specialist program must be a minimum of 6.5 years, which includes 1.5 years compulsory internship. Certain educational goals may necessitate a specified period of service in a learning environment for their attainment. These requirements are specific for each specialty.

National guidelines stress that ES involves reflection, advice, and ongoing support given by a skilled specialist who facilitates professional development and evaluation according to an educational plan (see Appendix 1). Regular meetings between residents and their educational supervisor are essential. The responsibility of assigning a dedicated ES supervisor to each resident rests with healthcare institutions managers.

### Data collection

Data was gathered in the period May to July 2023. When accessing the questionnaire, detailed information about the study was offered and participants had to give written consent for voluntary participation and data management before being allowed to proceed. To ensure unique answers, participants were asked to provide their e-mail address. The e-mail address could not be linked to the responses. We also introduced a selection question about what speciality program they were admitted to. This allowed us to exclude potential respondents from other health professions and specialities, who occasionally participates in the courses.

We collected data as written narrative responses, which involved study participants writing data in a qualitative questionnaire.^19^ In addition to questions about background variables, the questionnaire consisted of four broad guiding questions, allowing residents the opportunity to freely express their experience with ES (see Appendix 2). The following definition of ES was offered: planned, regular, and protected conversations between the resident and the supervising specialist to discuss various issues related to the education and the workplace where the learning takes place. By offering this shared point of reference, we aimed to reduce interpretive confusion and ensure that participants’ responses reflected experiences related to the intended concept.

We received a total of 108 responses. Twelve of the respondents were not applicable to our survey – answering ‘other specialities’ or ‘not being a resident’, – and therefore opted out of the survey. The responses from 96 participants were applicable for further analyses. In total, the data consisted of 28 pages of text using standard fonts. To enhance transparency and contextualize the data, key characteristics of the participants are described in Table 1 below.

**Table 1:** Demographics of participants

Theme	Subtheme	Meaning Unit	Summary of Meaning
Challenges in Implementing EPAs	Demanding Workload	Inability to Provide Immediate Feedback	Instructors may be unable to provide timely feedback to residents due to their heavy workload, leading to the potential loss of critical learning opportunities, as the rationale behind clinical decisions may be forgotten.
			Allocating sufficient time for feedback is essential, yet challenging, especially in high-volume clinical settings where patient care demands often take precedence.
		Demanding Workload	Teaching responsibilities can only be effectively carried out when the workload is manageable and the instructor is mentally prepared and focused.
			Balancing teaching duties with clinical responsibilities requires significant additional time and cognitive effort, which can contribute to instructor fatigue and reduce the overall quality of teaching.

### Data analysis

For our analysis, we used grounded theory based on Strauss and Corbin procedures: open coding, axial coding and selective coding.^18^ All authors read the elicited text from the survey to familiarise themselves with the data. Open coding was performed by the first author, using NVIVO. In this initial open coding phase, we looked for the meaning and dimensions of the data, generating codes that captured key actions, experiences and conditions. The research team met regularly to discuss and refine codes through constant comparison. During the axial coding phase, we organised codes onto higher-level categories based on their relationships and contextual connections. In the selective coding phase, we identified categories to explain the central phenomenon emerging from the data.

#### Reflexivity

Our collective experiences across pedagogical health science and medical fields were leveraged in this study. The first author (CNB) brings substantial expertise in evaluating residents’ training in hospital setting, the second author (KIR) is an experienced medical doctor with direct involvement in educational supervision of residents, and the last author (MK) has a background in higher education with a special focus on medical education and faculty development. This background was deemed crucial, both for developing the questionnaire and analysing the data in the context of residency training in a hospital setting.

All authors collaborated in creating the questionnaire. CNB recruited participants from national specialist courses, encompassing residents from various hospital specialties. All authors thoroughly reviewed the data material and held regular meetings to discuss interpretations of the material and to develop codes throughout all coding phases. CNB drafted the manuscript with help from the other two authors.

## Results

In the following, we describe and elaborate on the main categories demonstrating how hospital medical residents navigate between guidelines for best practice (see Appendix 1) in ES and resources embedded in their workplace culture. The overall impression of the material is that ES practices vary greatly: Whereas many residents do not receive ES at all, others report regular, well-functioning, and structured ES. Within this spectre, three main categories were identified: i) Access to educational supervision, ii) Links between quality of ES and organisational facilitation, and iii) Pushbacks to ES and how residents cope with these pushbacks.    

### Access to educational supervision

Almost all respondents reported that they were assigned a supervisor from the department upon employment. However, establishing contact between the resident and the supervisor could take a while depending on who took the responsibility for the first meeting. Once contact was made, some residents reported having regular meetings at scheduled times, whereas others met more sporadically, based upon either the supervisors’ or the residents’ request and availability. Subsequently, descriptions vary regarding whether the resident or the educational supervisor took the initiative to schedule meetings, one respondent stated: ‘Both the supervisor and I can take the initiative, but most often the supervisor' (No.96, Male, Anaesthesiology), while others described this shared responsibility as typically being delegated to the residents:

‘Everyone is assigned an educational supervisor and there is a shared responsibility between the supervisor and the resident; most often the responsibility lies with the resident.’ (No. 66, Male, Surgery)

Several residents described that ES meetings had either not taken place or that their educational supervisor had not contacted them to schedule a meeting:

‘Residents themselves have to take the initiative and find a time that is suitable, but this is often difficult. I have not received individual ES so far.’  (No. 50, Female, Internal medicine)

Many residents found it challenging to initiate ES-meetings, especially if the initial assignment of an educational supervisor was delayed:

‘I was not assigned an educational supervisor when I started as a resident in the department and had to ask for one myself. Eventually, I was assigned an educational supervisor, who only works at the outpatient clinic and is never present in my clinical everyday life...Several attempts to meet were forgotten before it actually happened.’ (No. 7, Female, Internal medicine)

Some residents described how they constantly had to request meetings. Although time was allocated for ES each month in their educational plans, actual meetings occurred less frequently. Residents often experienced having to reschedule meetings that for various reason were cancelled, stating: ‘Sometimes I wish that it was easier to get educational supervision, without having to work a lot to get a meeting with a supervisor.’ (No. 61, Female, Internal medicine) or ‘ES is organised only after I contact my educational supervisor several times. Then the educational supervisor chooses a suitable time. Then meetings are often cancelled because the educational supervisor has other conflicting tasks.’ (No. 14, Male, Internal medicine). Repeated cancellations were interpreted as a sign that ES was not a priority.

### Links between quality of ES and organisational facilitation

In the descriptions on how ES was conducted in practice, we identified three main patterns that in sum illustrate how the quality of ES, as experienced by the residents, was linked to organisational facilitation: residents receiving ES according to guidelines, residents experiencing deficient ES, and residents experiencing alternative types of supervision or lack of support.

#### Residents receiving ES according to guidelines

We identified a group of participants who described receiving ES in line with existing national regulation and guidelines: organised and structured with regular scheduled conversations in a dedicated setting. These residents described the content of a typical ES session as involving mutual responsibility between themselves and the educational supervisor. Residents in this group reported having regular ES sessions with their supervisor, on average every second month. Sessions were described as being tailored to residents’ needs, including feedback from educational supervisors on the potential for improvement, as well as a plan for continuing regular supervision:

‘I have a serious educational supervisor, so I mostly manage 10–11 ES sessions a year. We always plan the next ES session when the conversation ends, after about 45–60 minutes. Time is set aside in a private place. We systematically go through professional/learning goals, both professional and general, and discuss professional challenges/patient challenges, but also any collegial/healthcare institution demands. However, I believe this is a strong deviation from what my colleagues experience [where many struggle to meet their supervisor regularly and experience deficient ES].’ (No. 16, Male, internal medicine)

Furthermore, residents in this group described ES as a safe space for discussing their well-being and personal or difficult matters. One participant summarised it this way:

'During ES, I talk to the supervisor about progression in educational courses, about my strengths and weaknesses, well-being in the department, etc. Learning objectives that involve topics to be discussed during ES. We create the agenda as it comes – what is needed right then and there. I feel that I benefit from ES.’ (No. 41, Female, Internal medicine)

Overall, the participants in this group expressed satisfaction with ES and their suggestions for improvement of ES were concretely about topics they wanted to talk more about in the ES situations.

#### Residents experiencing deficient ES

A second group was comprised of residents who reported having regular, but deficient ES.  They reported inadequacies related to the organisation of ES and the specific content in ES meetings. Suggestions for improvements included clearer guidelines on the expectations for residents and educational supervisors, as well as better routines and structures for implementing ES. They wanted more concrete personal feedback and less focus on the administrative demands attached to documenting and assessing learning outcomes. Although the ES structures seemed to meet the formal requirements, the respondents experienced that it did not correspond with their expectations of ES.

‘Unfortunately, the educational supervisor had little knowledge of how educational supervision should take place, and we don’t talk much about difficult things.’ (No. 14, Male, Internal medicine)

Residents described that the agenda of ES meetings was often unclear, or that the educational supervisors lacked insight into the residents’ competence. As a result, residents gained little benefit from the conversations:

‘At the last ES session, we talked about my progress, which the educational supervisor has only heard from me, he/she had not taken the time to ask other senior doctors who work with me. In summary, ES is quite absent and of poor quality.’ (No. 7, Female, Internal medicine)

Participants in this group shared their perceptions on the quality of the ES sessions. They were also able to articulate their needs and expressed uncertainty about whether ES contributed to their learning. Several expressed that they wanted more commitment from ES supervisors and more active involvement in ES sessions:

‘Missing a presence in ES and closer follow-up, want allocated time for ES at least twice a year.’ (No. 71, Male, Surgery)

#### Residents experiencing alternative types of supervision or lack of support

One group reported finding ES so unreliable or unhelpful that they sought out alternative support. Some residents noted a lack of organisation in scheduling ES, as it was not always aligned with the individual work plans of either residents or educational supervisors. This would often result in educational supervisors or residents being off duty when ES was scheduled, with cancellation, rescheduling or additional efforts on the resident’s side to make ES happen:

‘Several times, I came in to work on my days off to complete supervision sessions.’ (No. 57, Female, Internal medicine).

Furthermore, the lack of structure in ES meetings and the number of conflicting tasks made it difficult to prioritise ES. Others claimed their supervisor was unprepared, resulting in conversations that were unspecific and unstructured. Residents reported being left with a feeling that their educational supervisor lacked sufficient knowledge to be able to provide useful feedback. One participant described this:

‘No one has prepared a topic. The last time was an ad hoc meeting in the Emergency Department when both were on duty.’ (No. 12, Female, Internal medicine)

The participants expressed that they wanted more focus on relevant topics connected to the work environment and demanding situations that they experienced. They also expected more regular and formalised ES meetings, along with better availability of ES to monitor their progression.

A final group of residents experienced a total lack of ES. Even though a formal ES supervisor was appointed, they still reported that they ‘Never had an ES session’ (No. 95, Female, Surgery) or ‘We haven’t had a single ES in almost four years’ (No. 70, Male, Surgery). Some elaborated on the reasons for its absence, claiming it is ‘due to lack of a consultant present. Educational supervisor had been away for over a year.’ (No. 27, Female, Internal medicine). Due to the lack of experience, residents in this group had little to say about ES. They expressed no expectations for what the content of ES meetings should be and focused instead on obtaining ES at all.

### Pushbacks to ES and how residents cope with these pushbacks

Many residents experienced that planned ES meetings were repeatedly not prioritised, that they were cancelled, or rescheduled. These pushbacks hindered them from accessing ES. They then seemed to compensate by adopting strategies that gave them some form of alternative support. Two subcategories were prominent within this category: one describing pushbacks to ES and one focusing on residents’ strategies for dealing with pushbacks.

#### Pushbacks to ES

Pushbacks became apparent when participants experienced barriers to accessing ES meetings. These barriers included initiatives being ignored or constantly needing to ask to reschedule cancelled appointments. Other examples of pushbacks were lack of continuity in organising ES and lack of engagement and commitment from an educational supervisor. As one participant noted, there was a sense of: ‘missing an educational supervisor who is motivated to give ES’ (No. 7, Female, Internal medicine). Another participant described how ES was ‘Difficult to achieve due to the service schedule, [..] which means that you are either not at work at the same time as your supervisor or you simply do not have the time.' (No. 65, Male, Surgery).

The residents described that structures originally designed to provide support, such as documenting the sessions, could unnecessarily complicate ES and hinder its implementation. Others described time as a crucial condition and that administrative work attached to ES (scheduled meetings, writing minutes, reporting attendance, etc.) added additional workload. One participant described this as follows:

‘There are too many administrative requirements around ES. It complicates things unnecessarily. There is much better supervision in what happens in ad hoc situations.’ (No. 30, Female, Internal medicine)

Consequently, residents experienced distance from their educational supervisors when these demands took too much space during the ES session, which in turn had a negative impact on the relation between resident and educational supervisor. One participant described a typical ES session like this:

‘I was involved in a clinical situation which has subsequently led to thoughts about how it could have been resolved better. Then we talk through the various assessments that were made and why. In other words, like a debrief with a complete outsider.’ (No. 94, Male, Anaesthesiology)

#### Residents’ strategies for dealing with pushbacks

Residents who either experienced a lack of ES, infrequent or low-quality ES developed compensating strategies to get the support they needed. For some, getting a new educational supervisor helped: ‘After a change of educational supervisor…the topics and structure around the ES session became more systematic.’ (No. 81, Male, Internal medicine). Others developed strategies like asking for support and advice from other colleagues:

‘I rarely get ES from my educational supervisor, but I often get ES from other senior doctors. I may more often use those I believe I have a good relationship with...My last ES session was a few weeks ago, but not with my educational supervisor.’ (No. 68. Female, Surgery)

One participant described how ES was neglected, forgotten, and downscaled, which led to attempts to compensate by relying more on clinical supervision instead of ES:

‘I don’t receive educational supervision. It’s more like clinical supervision. Throughout my residency training, I had to make sure, on my own, that I had an overview of learning objectives and how I could achieve them. Unfortunately, supervisors have not received training in this.’ (No. 47, Female, Internal medicine)

Participants who were not given the opportunity to have ES expressed a sense of resignation in not knowing whether it would have been useful for their education. Some adopted the mindset they were met with from the educational supervisor or department, where ES was not prioritized. They resigned, expressing themselves the attitude that ES was not important:

‘I feel that I do not particularly benefit from the ES. It’s more like a cozy coffee chat, than anything else.’ (No. 14, Male, Internal medicine)

## Discussion

The results from our study highlight significant variations in how ES is organised and in who takes responsibility for ES being carried out in different departments. We found descriptions of how lack of routine for organising ES led residents into energy draining pathways where ES was planned, cancelled due to lack of time or competing tasks, and then rescheduled often on the residents’ initiative. These efforts can result in a completed ES session, a new postponement, or to resignation, as ES is perceived as being unattainable or unimportant. Some residents relied solely on irregular ES meetings taking place upon request from the residents themselves.

Ensuring that residents have access to ES is important,^21^ but the content of the sessions is also vital for them to find it useful. Previous studies have highlighted that accessible supervisors were essential in ensuring meaningful content in ES sessions, while time constraints posed a challenges to its organisation.^10^ Robbrecht and colleagues emphasised that, while supervisors could influence and optimise time spent in the ES setting, the allocation of time for ES was often beyond their control.^8^ Our data revealed three distinct groups of residents with varying experiences of ES, each demonstrating different opportunity levels of engagement from management and supervisors. There was also varying resources available within each group, such as available time and organisational structures surrounding ES.

The use of compensatory strategies suggests that residents are actively taking charge of their educational journey. This, in turn, can be interpreted as an indication that they recognize the need for ES. The opportunity for residents being able to express their needs and have them met is significant for learning.^22^ However, there is an inherent complexity to residents’ requesting support which has been explored in previous studies.^11^ Kennedy et.al found that the resident perceives seeking support from supervisors as a potential threat to their professional credibility.^23^ For residents, the ability to open up and be vulnerable with someone who shows interest in their growth, invests in their learning, and listens attentively is essential in the supervisory relationship.^24^ Conversely, when trust levels are low, residents are less likely to share their vulnerabilities. Additionally, Birkeli and colleagues noted that educational supervisors observed that residents’ struggle to openly discuss time pressures, work-home balance, and the pressure to perform without revealing vulnerabilities in ES sessions.^13^ This balance between educational and personal support was identified as a challenge for educational supervisors.

Put into a wider context, previous studies indicate that the workplace environment influences how residents weight the decision to seek support or not, thus framing their help-seeking as a balancing act.^11^ Therefore, engagement and prioritisation from management are essential. With the implementation of CBME in Norwegian medical residency training in 2019, residents’ right to ES was formalised and guidelines describing responsibilities, ES frequency, and recommended approach were developed by the Norwegian Directorate of Health.^25^ Earlier studies show that implementation of changes in a healthcare system can be complicated and require a strategic vision and communication across different organisational levels.^26^^,^^27^ When ES is not given priority at a management level, the responsibility shifts to individuals, such as educational supervisors and the residents. As a result, educational supervisors are left alone with the responsibility, ^13^ while residents feel they must constantly request ES.

Establishing educational structures that support effective ES in practice is essential, however they must be feasible within the existing health care system where the educational practice occur. Our findings illustrate the importance of having formalised structures and responsibilities securing residents’ access to ES, but also that establishing guidelines and requirements on paper is not enough to guarantee either that ES takes place or ES of high quality. We need to approach ES in a manner that allow residents to concentrate on specific topics, tasks, feedback, and advice that are beneficial for their education and growth.^14^

### Limitation

Although this study was based on narrative data from a large number of relevant respondents, the findings are limited by the lack of in-depth data that could have been obtained through interviews, where additional probing and follow-up questions from researchers might have yielded richer and more detailed insights.^18^ The broad prompts allowed respondents to articulate their most pressing issues concerning ES, in their own words. However, the use of open-ended questions may have limited the identification of certain themes such as Equity, Diversity, and Inclusion (EDI), which did not surface in our findings. Furthermore, although we provided residents with a definition of ES in the questionnaire, we acknowledge that ES remains poorly understood and that local understandings may have superseded our definition and influenced the responses. Consequently, our contextual interpretation of the data may have affected how we translated excerpts from Norwegian to English.

### Implications for future practice and research

Findings from this study indicate that many residents struggle to engage with ES in ways they perceive as meaningful, suggesting a need for more robust, structured, and routine systems at the management level for organising and conducting ES sessions. When such conditions are lacking, residents often bear the burden of having to engage in energy-draining initiations of ES. Educational leaders can assist supervisors in assuming mutual responsibility by providing structured, scheduled, and well-organised frameworks for ES that are practical and feasible for both residents and supervisors. As highlighted in other studies, educational supervisors must be trained in ES and be given time to be accessible, as they play a crucial role in supporting residents’ growth and learning.^2^^,^^6^^,^^13^^,^^28^ Finally, the learning needs of residents should be recognized, which affords trusting relationships and open communication between residents and educational supervisors where residents feel comfortable seeking help and support.

Further research is needed to explore affordances relevant for different medical specialties. In addition, observational studies are much needed as a complement to self-reported data to deepen our understanding of how ES, residents, supervisors and clinical duties interrelate in the workplace environment. The role of educational leadership in CBME implementation also warrants further exploration.

## Conclusion

In this study we explored how hospital residents engage with ES in the context of CBME implementation. Our analysis suggests that the attainability and benefit of ES, as experienced by the residents, is shaped through a dynamic interplay of factors, including residents’ own initiative, supervisors’ knowledge, skills, and attitudes toward ES, the prioritization of ES by management, and the affordances of the workplace environment. These elements emerged as interrelated conditions that influence how ES is experienced and enacted in practice. Proper organisation and follow-up of ES for residents was found to play a critical role. When ES is well-structured and integrated into clinical practice, residents appear better able to articulate its benefit to their education. Conversely, inadequate organisation of ES forces residents to expend significant effort to ensure meetings occur. While learner’s initiative and agency are to be expected, persistent lack of reciprocity seems to hamper full engagement with ES. Amidst the implementation of CBME, residents risk being left with the individual responsibility to initiate and sustain ES, which in turn places an undue burden on trainees to navigate repeated pushbacks, and workplace cultures that devalues educational support.

### Conflict of Interest

The authors declare that there is no conflict of interest.

## Supplementary material

Supplementary file 1 Appendix 1. Summary of national guidelines on educational supervision from the Norwegian directorate of health

Supplementary file 2 Appendix 2. Guiding questions used in the questionnaire
