# Supplementary material for: Scheduled, cancelled, rescheduled: navigating educational supervision in residency training
Source: Int J Med Educ. 2025 Jul 28;16:128–37. doi: 10.5116/ijme.687b.7d22 (PMC12700665; doi:10.5116/ijme.687b.7d22)
Supplement: Supplementary file 1 — Appendix 1. Summary of national guidelines on educational supervision from the Norwegian directorate of health [file ijme-16-128-S1.pdf]

Appendix I

Summary of national guidelines on educational supervision from the Norwegian directorate of health

**Educational supervision is in the guidelines defined as** *"Reflection, advice, and follow-up on professional progress during specialization, and contributions to the assessment of residents. Educational supervision involves planned and regular meetings between the resident and the supervisor. The meetings shall be kept separate from other work tasks."*		
**The role and tasks of the organisation/leader regarding educational supervision**	**The role and tasks of the educational supervisor**	**Content of the educational supervision**
3:1 The leader in the educational institution shall appoint an individual supervisor for each resident. The supervisor shall have necessary competence.<br>3:2 The leader in the educational institution should ensure that the supervisor is known to the resident as early as possible upon employment.<br>3:3 The leader of the educational institution should, as soon as possible after a resident's employment, inform the educational supervisor about the resident(s) they will be supervising.<br>3:4 The leader in the educational institution shall ensure that the educational supervisors and resident have sufficient allocated time for educational supervision.<br>3:5 The leader should facilitate continuity in educational supervision, and the resident should, at a minimum, have the same supervisor throughout a specialisation period.<br>3:6 The leader in the educational institution shall ensure that the educational supervisor is informed about how responsibilities, tasks, and authority related to educational supervision are organised and distributed within the institution.<br>3:7 If the task of approving learning objectives is delegated, it is the leader's responsibility to ensure that a sufficiently neutral assessment of the resident is given, and that the educational supervisor does not face conflicting roles.<br>3:8 The leader shall seek advice from the educational supervisors on the assessment and formal approval of all learning objectives.	4:1 The educational supervisors shall familiarise themselves with the learning activities and assessment methods specified in the specialty's educational plan.<br>4:2 The educational supervisors shall familiarise themselves with the educational path the resident is undertaking and assist in developing an individual educational plan.<br>4:3 Individual educational supervision sessions should be held at least every fourth week, lasting 45-60 minutes.<br>4:4 Within family medicine, community medicine and occupational medicine educational supervision should be planned and held regularly.<br>4:5 The supervisor shall initiate the first educational supervision meeting, which should take place no later than three weeks after the start of residency.	5:1 The educational supervisor shall provide residents with professional guidance through reflection on relevant learning objectives.<br>5:2 The educational supervisor shall assess the residents' competence related to all learning objectives (both subject-specific learning objectives and general competency objectives).<br>5:3 The educational supervisor shall, together with the resident, assess the need for changes in the individual educational plan and provide advice to the residents' leader on this.<br>5:4 Each resident is responsible for their own learning, including actively contributing to the educational supervision meetings.
