# Supplementary material for: Scheduled, cancelled, rescheduled: navigating educational supervision in residency training
Source: Int J Med Educ. 2025 Jul 28;16:128–37. doi: 10.5116/ijme.687b.7d22 (PMC12700665; doi:10.5116/ijme.687b.7d22)
Supplement: Supplementary file 2 — Appendix 2. Guiding questions used in the questionnaire [file ijme-16-128-S2.pdf]

Appendix 2

Guiding questions used in the questionnaire

We seek information about your experiences, thoughts, and descriptions of how the educational supervision you receive (or do not receive) works for you. In the following questions, we have provided guiding questions for each section, but if there are other aspects of supervision you wish to share that are not covered by our guiding questions, please feel free to share them.
By "educational supervision" we mean planned, regular, and protected conversations between the resident and the supervising specialist to discuss various issues related to the education and the workplace where the learning takes place.
1. How is the supervision organised and how often do you receive supervision? Who takes the initiative for the meetings? Can you describe a typical (or your most recent) supervision meeting? *[please write here*]
2. What do you talk about in the meetings? Who sets the agenda? What do you gain from the educational supervision? *[please write here*]
3. How would you describe your relationship with your educational supervisor? What do you expect from your supervisor? *[please write here*]
4. Is there anything else you would like to share about the educational supervision? Is there anything you feel is lacking in educational supervision? *[please write here*]
